# Research priorities during infectious disease emergencies in West Africa

**DOI:** 10.1186/s13104-018-3263-3

**Published:** 2018-03-01

**Authors:** Morenike Oluwatoyin Folayan, Bridget Haire, Dan Allman, Aminu Yakubu, Muhammed O. Afolabi

**Affiliations:** 1 Institute of Public Health, Obafemi Awolowo University, Ile-Ife, Nigeria; 2 Kirby Institute, University of New South Wales, Sydney, Australia; 3 Dalla Lana School of Public Health, University of Toronto, Toronto, Canada; 4 Federal Ministry of Health, Abuja, Nigeria; 5 Department of Clinical Research, London School of Hygiene & Tropical Medicine, London, UK

**Keywords:** Infectious disease, Epidemic, West Africa, Research priorities, Research design, Consultation

## Abstract

**Objectives:**

This paper presents the results of the consultations conducted with various stakeholders in Africa and other experts to document community perspectives on the types of research to be prioritised in outbreak conditions. The Delphi method was used to distill consensus.

**Results:**

Our consultations highlighted as key, the notion that in an infectious disease outbreak situation, the need to establish an evidence base on how to reduce morbidity and mortality in real time takes precedence over the production of generalizable knowledge. Research studies that foster understanding of how disease transmission could be prevented in the future remain important, implementation research that explores how to mitigate the impact of outbreaks in the present should be prioritized. Clinical trials aiming to establish the safety profile of therapeutic interventions should be limited during the acute phase of an epidemic with high fatality—and should preferably use adaptive designs. We concluded that community members have valuable perspectives to share about research priorities during infectious disease emergencies. Well designed consultative processes could help identify these opinions.

**Electronic supplementary material:**

The online version of this article (10.1186/s13104-018-3263-3) contains supplementary material, which is available to authorized users.

## Introduction

The scale, duration and magnitude of human suffering experienced and witnessed during the recent West African Ebola epidemic had considerable impact on how infectious disease emergency responses are framed. Several trials to find candidate drugs to treat and vaccines to prevent Ebola were conducted as a result of this epidemic. However, none of the candidate drugs were found to have the required efficacy for treatment; and only one of the vaccine trials produced results pointing towards effectiveness against Ebola Virus Disease (EVD) [1]. An important lesson learnt, however, was that trials that meet internationally accepted standards can be conducted during disease outbreaks including those caused by high-hazard pathogens [1]. However, these studies had some contentious elements.

From a research ethics perspective, there are concerns about the social value of conducting research in such situations considering the high mortality and morbidity associated with EVD [2, 3]. The concerns relate to the social value of conducting such studies during a highly fatal infectious disease epidemic. Together, these concerns ignited debates on the justification for the conduct of randomised placebo-controlled trials (RCTs), prioritisation of resources, compassionate access to unapproved therapies, and the balancing of research and public health action [4–6]. Responding to these concerns, several articles were written including normative commentaries [7], empirical research [8]; and substantive guidance documents were developed [9–12] by researchers and public health practitioners alike. Researchers also discussed how the weak healthcare systems, inadequate healthcare resources, and the histories of civil war and political violence affected the epidemic responses [13]; and the impact of these on the choice of clinical trial design [4]. The challenge of providing appropriate ethics review and oversight to ensure ethical conducted of the studies in the region, under these circumstances, was also highlighted [14, 15].

Our work aimed to contribute to the discourses and efforts at addressing concerns of conducting research studies in disease outbreak situations. Overall, our focus is to develop a community engagement framework that draws both on the perspectives, empirical findings and guidelines developed so far, coupled with the experience of stakeholders who lived through the epidemic. In this paper, as part of our overarching work, we present community perspectives on clinical trial design and how these might inform future research during outbreaks. We report on the outcome of an extensive consultative process with experts in bioethics, community engagement, and research—including those involved with Ebola clinical trials in West Africa—social scientists and members of ethics committees in West Africa. The key outcome of this consultation was a consensus statement on research priorities and appropriate clinical trial designs during such epidemics.

## Main text

The consultative process used the Delphi method to reach a consensus [16–19]. It allowed for a group process that involves a series of iterative interactions with experts on a complex issue of interest to various stakeholders—bioethicists, social scientists, ethics committee members and community members—with no history of conclusive decisions. The Delphi technique had been used in the past to reach consensus about issues related to randomized controlled trial design [20, 21].

There were three phases of iterations and an additional round of iteration each for phases I and II. Phase I involved a face-to-face consultation with eight research experts, bioethicists and community engagement specialists to answer four research questions. Phase II involved a review of the document developed in Phase 1 by three experts—a bioethicist, a researcher involved with clinical trials for Ebola vaccine evaluation in West Africa, and a researcher working on community engagement issues in Africa. A second iteration included these three experts and the eight experts involved with Phase I. A consensus document was developed which included only issues where consensus was reached. Phase III involved consultative discussions on the consensus document developed in phase II with 20 members of the Network of Ethics Committee members in West Africa. They (dis)agreed with the statements in the consensus document, and discussed their views during a plenary session. MOF collated and analysed the themes and sub-themes that emerged from all the iterations (see Additional file 1).

### Research priorities

Participants that took part in the consultations posited that during an infectious disease outbreak, research studies that focus on mitigating suffering in the present and those that seek to identify means of prevention in future epidemics are those that need to be prioritised. Where there are various unknowns and uncertainties about the utility of existing medications and the existing clinical care pathways and systems to cope with an epidemic, implementation research should be prioritized during the acute phase of an epidemic with high fatality. Implementation research was understood as research to explore improved or novel ways to address disease conditions including clinical care pathways and health systems challenges that would provide affected persons with the best chances of survival. While undertaking any of these research studies, participants argued that researchers should use designs that increase the prospect of including affected populations as study participants. However, while included in such studies, the ideal design should also make it possible for the participants to access therapy (with known or unknown efficacy, when or where available).

Participants noted that even in an emergency disease outbreak, the act of research alone could not be construed as an emergency undertaking. It could be valuable, arguably even necessary, but could not be constituted as an emergency per se. Thus, unless the purpose is to determine the efficacy or effectiveness of a clinical response to an intervention including drugs with a significant likelihood to benefit patients, clinical trial studies need not be a priority during the acute phase of an infectious disease epidemic with high mortality and morbidity. Limiting clinical trials in this manner would avoid distraction from optimal use of limited resources—human especially—that should otherwise be invested in the public health response. In effect, in the acute phase of the infection, phase II and III drug trials could be prioritized over those that aim to establish the safety profile of therapeutic interventions (phase I studies). Clinical trials that do not diminish the prospect for morbidity and mortality arising from the infectious disease emergency should not be prioritized.

### Research designs

The consensus of opinions during the consultative process was that research conducted during a self-limiting infectious disease epidemic should be designed in a way that is flexible enough to address immediate community needs of decreasing mortality and morbidity. Participants argued for the use of adaptive trial designs [22] to test interventions, in order to increase the prospect of making timely changes to trial designs, as may be required. Where there is a public health response that supports the compassionate use of research products, the research design should not exclude the use of such drugs.

Participants had strong views about equity of access to potentially useful interventions, even where such interventions are not proven to be safe and effective. Where an epidemic affects children and pregnant women, clinical trials should be planned in a cascaded fashion that allows for the recruitment of children and pregnant women during different waves of the trials. This way, as data on safety becomes available, enrolment of these populations with particular vulnerabilities can be considered. In order to facilitate this, research teams should consider inclusion of children and pregnant women in research studies early during the research concept development stage.

During infectious disease outbreaks, the main motivating factor for research participation is not altruism. Altruism—the decision to enrol simply for the benefit of others—has been documented as one of the top motivations for participation in research studies in other contexts. However, participants from our consultations held that where there is intense fear and uncertainty as in the case of the Ebola epidemic, people choose to participate in research as a health-related resource-seeking strategy. Individuals who volunteer as research participants during infectious disease emergencies with high morbidity and mortality, with little or no prospect for research-related benefits, may assume that the State would take care of their needs in the face of any eventualities. Researchers need to recognise these realities of the lives of study participants, and work with appropriate authorities—community members and research ethics committees—to define appropriate compensation including insurance cover for research participation in these situations.

### Discussion

Participants believe that in an infectious disease outbreak, the need to produce generalizable knowledge is subordinate to the need to establish evidence on how to reduce morbidity and mortality in real time. While conducting research that would have a greater benefit in the future was seen as important, it was less so than providing a robust response to patient care and outbreak control during an on-going infectious disease epidemic. This was why participants felt that where clinical trials are considered for implementation during an outbreak, adaptive trial designs should be prioritised. This would allow for modifications of the trial and/or statistical procedures of the trial after its initiation without undermining its validity and integrity. It could permit liberalised access to unlicensed drugs through compassionate access; and accommodate inclusion criteria that prioritise the broadest access for those at risk.

This view—prioritising amelioration of suffering in the present over saving future putative lives—contrasts with argument put forward by Eyal and Lipsitch [23], that traditional RCTs should be prioritized because they could save more lives in the longer term by virtue of the greater certainty they provide about an intervention’s level of efficacy. Doussau and Grady [24] in their critique of the ‘stepped wedge design’ had opined that hope is a poor basis for science albeit in an attempt to argue against the potential of the design to allow participants receive the intervention at some point. Our findings however suggest that hope might be an important community value that could help foster and maintain support for clinical research and public health interventions during an epidemic of crisis proportions.

Considerations for ensuring timely data generation for women and children through the cascading of clinical trial findings that fast-track their recruitment, may be suggestive of the need to review current ethical guidelines on recruitment of women and children in clinical trials specifically during emergency outbreaks. While a Delphi consultation may not provide sufficient evidence to justify this major change in current recruitment practices for clinical trials, this finding should inform on-going deliberations on alternative clinical trial designs conducted during emergencies.

Also, the principle of reciprocity [25] requires that communities hosting research should be left with long-term research associated benefits where feasible. Investing resources and prioritising research that results in the development of appropriate and effective supportive packages of careduring infectious disease outbreaks is a way to ensure communities are left better off after the conduct of the research. Infected and affected people may receive immediate benefits from intervention research; and future patients will also benefit from the research outcomes. During the last Ebola epidemic, not enough was learnt about the true benefits (or potential harms) of administering specific supportive care packages appropriate for resource-limited settings [1, 26, 27]. Rigorous reviews of existing data in order to codify a decent, evidence based standard of care are urgently required.

## Limitations

The use of Delphi as a consultative method has the risk of discussants reaching a compromised consensus with some of the points of dissent and contention, and the reasoning behind these getting lost [16]. The consensus reached are also based on the constructed reality of the experts consulted thereby limiting its fit into the traditional reliability and validity criteria [19]. The process however helped us identify some of the common values held by those consulted. Also, discussions about study designs included perspectives of lay-persons who had limited expertise and experience with clinical trial design. It is possible that if trained scientists were involved in the consultation, the discussions on appropriate research design may have been broader. Further, our participants were willing to support in theory, increased risks with regard to earlier participation by pregnant women and children rather than exclusion, a perspective that challenges current ethical norms; and might be defensible with respect to an outbreak as devastating as Ebola. This risk tolerance might change with less deadly epidemics.

## Additional file

**Additional file 1.** Ethical considerations and community engagement when conducting clinical trials during an infectious disease emergency in West Africa. A collation of the consensus report during the phase 1 to IV consultative process. The document had been arranged into themes and sub-themes that emerged from all the iterations. Also, outstanding questions generated during the process were collated.

## Abbreviations

RCT: randomised controlled trial
WHO: World Health Organisation
