# Supplementary material for: Research priorities during infectious disease emergencies in West Africa
Source: BMC Res Notes. 2018 Mar 1;11:159. doi: 10.1186/s13104-018-3263-3 (PMC5831857; doi:10.1186/s13104-018-3263-3)
Supplement: Supplementary file 1 — Additional file 1. Ethical considerations and community engagement when conducting clinical trials during an infectious disease emergency in West Africa. A collation of the consensus report during the phase 1 to IV consultative process. The document had been arranged into themes and sub-themes that emerged from all the iterations. Also, outstanding questions generated during the process were collated. [file 13104_2018_3263_MOESM1_ESM.docx]

ETHICAL CONSIDERATIONS AND COMMUNITY ENGAGEMENT WHEN CONDUCTING CLINICAL TRIALS DURING AN INFECTIOUS DISEASE EMERGENCY IN WEST AFRICA

Bioethics policy maker Expert input received: 19^th^ November, 2017

Community expert input received: 21^st^ November, 2017

Community stakeholder/policy maker expert input received: 8^th^ December, 2017

1. **Introduction**

The outbreak of Ebola Viral Disease (EVD) epidemic, which mostly affected the West African countries of Sierra Leone, Guinea and Liberia; and also affected Nigeria, Senegal, Mali, United States, and Canada, led to various initiatives aimed at strengthening the capacity of countries and the global community to respond better in the future to epidemics of such magnitude of mortality and morbidity. One of the key issues of concern was how to conduct research during such emergency situations. In May 2015, two important World Health Organisation led consultations took place – one of the two summits on research and development examined ways to build collaborations to accelerate access to novel interventions in emergency situations. The other was a consultation to develop a guidance document for managing ethical issues encountered during infectious disease outbreaks. The guidance document, published in 2017, highlighted the role of community in infectious disease outbreak response, as well as research under such situations. The document posited that “*all aspects of infectious disease outbreak response efforts should be supported by early and ongoing engagement with the affected communities. In addition to being ethically important in its own right, community engagement is essential to establishing and maintaining trust and preserving social order*.”

Our work builds on the guidance document, taking on the subject of community engagement in greater depth in order to propose far-reaching and more expansive ethical considerations for community engagement, specifically when conducting research during infectious disease emergencies. First, we conducted an online survey seeking public opinions from lay persons on their perspective of critical elements of a response during infectious disease epidemics. We plan to write up the results of this survey at a later date as a manuscript. We shall compare the perspectives shared and those highlighted n professional documents published in the literature and in World Health Organisation guidance documents.

Next, we held a symposium in Abuja, Nigeria on the 14^th^ and15^th^ December, 2016 with eight key experts on bioethics and community engagement (CE) from Nigeria, Australia, Liberia, and Canada. We discussed the following key questions and pooled together recommendations – a) what should/ought to be the objective(s) of community engagement during an infectious disease emergency?; b) how should Community engagement be conducted in such settings?; c) what are/should be the key community engagement considerations during research design and implementation; d) in what ways does the history, political and socio-cultural contexts affects community engagement in research conducted during emergency?; and lastly, we discussed e) Community omission – if it ever is justified and under which conditions would it be justified to omit any form of CE in such studies. Members present at the consultative meeting also had access to the outcomes of the online survey.

The experts at that face to face meeting held on the 14^th^ and 15^th^ of December, 2016 also had the opportunity to provide a second round of inputs to the document once a summary document was developed. They had access to the summary document and made their second round of inputs between 12^th^ and 19^th^ of August 2017.

The meeting outcomes were shared with three other experts who could not make the meeting physically for their inputs. One of the expert had worked extensively in the field of community engagement in research and is resident in Kenya. The other is a researcher and bioethicists who currently works with Ebola research in Sierra Leone. The third was a bioethicist from Nigeria. They provided feedback to the document between 24^th^ of August and 4^th^ of September 2017. Their perspectives also feed into the final outcome document developed from the consultative meeting.

Next, at a consultative meeting of ethics committee members from the regions affected by Ebola in West Africa, held on the 25^th^ to 27^th^ of September, 2017 in Senegal, 20 persons – bioethicists, researchers, social scientists, an ethics committee desk officer and laypersons – who work with ethics committee in the region and/or are involved with review of protocols during the Ebola epidemic, discussed the outcomes and recommendations from the consultative meeting. This was to further validate the key highlights of our Abuja symposium.

The notes below form a documented summary of the highlights of the Abuja deliberation, the comments received through the iteration processes, and the Senegal deliberation. We are sharing this note with you as external validation experts. We plan to share with experts who had been involved with development of community engagement and bioethics guidelines. Thereafter, the reports from the engagements will be synthesized into one as the final grant report, while manuscripts will be written from the outcome of this consultative process subsequently. All contributors to this process will be duly acknowledged. Please feel free to move things around. Please ensure all your edits are made in track changes.

**2.0. Objective of community engagement during clinical trials conducted during infectious disease emergencies**

West Africa is a communitarian society. The community is an integral component of society and must be regaded as such. Community can be defined in many ways – geographic, patient group, demographic characteristic, political or cultural interests. For research conducted during epidemics we often refer to a geographic community in the first instance (the people potentially affected), which may of course have many smaller geographic and other types of communities involved, particularly where epidemic is spread over a country or sub-region.

One of the objectives of community engagement during clinical trials conducted during infectious disease epidemics is to facilitate the successful implementation of the research by building trust, enhancing public health education, promoting collaboration, and dispelling unfounded fear and rumours. Community engagement during infectious disease emergency also helps to ensure that the research is locally responsive – that the aims and processes involved are not only acceptable but serve the interests of people locally. It should also maximize benefits and minimize costs/burdens to participants and communities through understanding of how these are likely to work out in practice in a given setting (e.g. avoid unexpected/ perverse outcomes). It should also strengthen science by making sure that research tools are appropriate and the implementation process will likely result in collection of valid information.

- The goal of community engagement for the researchers is to ensure buy-in of community stakeholders in the planning and implementation of research to ensure success and minimize challenges, misconceptions and fuelling of rumours that jeopardise research. However, for community advocates – intermediaries between researchers and communities – the goal of community engagement is to ensure the protection of the rights and integrity of the citizens they represent, including ensuring the research addresses their needs.
- The ultimate goal of community engagement is to facilitate ownership of the research. Community engagement tends to stimulate interest as members accept the community response as their own home-grown efforts/contributions to their address the peculiar health related challenges affecting members of the community. It also tends to guarantee sustainability of the community response and minimize unhealthy rivalry for leadership positions within the research enterprise. This unfortunately is not the goal of many clinical trials conducted as joint ownership (with implications for intellectual property rights) implies shared goals, shared decision making and shared profits. These are not envisioned when clinical trials are being planned and conducted. For clinical trials conducted during an epidemic, the envisioned goal should be that of collaboration.

Public health response is not synonymous with research response. The rights of communities are limited by the laws that govern public health response. There are no laws that define the limit of community engagement in research. Often research is less urgent, and less demanding of immediate, rapid response and action. Efforts supportive of research implementation – inclusive of the community engagement process - should therefore not distract from the public health response instituted to contain the epidemic.

- The global nature of an epidemic should not exclude the practice of community engagement in research.

Community engagement helps to make science less paternalistic as it recognizes community as a strong and essential player in the research enterprise with as much relevant knowledge and skills. Space needs to be created for both community representatives and researchers to collaborate and exchange ideas so as to improve the outputs of science. We recognize that models developed for community engagement with the objective of addressing the paternalistic nature of science – focused on advancing social equity, inclusion, and wellbeing - may meet with implementation hitches during an emergency in societies where respects for rights of persons are not institutionalized as is the case with countries in West Africa.

- It is also important to recognize that community engagement plans may be specific to communities. When there is an ethical lens focused on community engagement practices, the paradigm that usually evolves is based on social practices and norms within that community. When a study is implemented by a research partner that has built a trust relationship with the community, the dialogue that ensued between the partners can lead to a shift in social and cultural positions based on an acknowledgement of the validity of the others’ position. The aspirational ideals of community engagement plans designed for a trial that is socially responsive is often particular to specific communities. The plan feeds into a social mobilisation process. It evolves from perception analysis of communities, social science, and formative research.
  1. **New Questions**
- What is successful community engagement?
- Would the objective of community engagement differ if PI is local vs if the PI is international?
- Is community engagement critical in different ways for different societies? Is community engagement a critical issue for communitarian societies and not that crucial in individualistic communities? If so, how does it differ and under what condition(s).
- What is the objective of community engagement in infectious disease research - is it to prevent failure of the research or to enhance uptake of the research outcomes? Or both? What else? How do you define failed research?

**3.0. How should community engagement be conducted?**

- Our model for community engagement when planning to conduct clinical trials during an infectious disease epidemic is one built on a model that facilitates collaboration between researchers and the community rather than one that promotes partnership. We acknowledge the limited ability to promote the goal of partnership for research conducted in West Africa knowing that most clinical trials are funded externally. We do not suggest that partnership should not be the target of community engagement plans. We however consider this an aspirational goal in the current context and climate of clinical trial practice in West Africa.
- The community engagement process should reflect integrity on the path of researchers with efforts at being respectful, fair, transparent, and accountable. Researchers, irrespective of nature of the epidemic, need to be accountable to the public and the community. The research governance structure, including the research ethics committees that often act for the community, should ensure that the research process reflects these values.
- Researchers judge competency of the community based on the standards enacted by them. Researchers require communities to measure up to those standards when they are invited/brought to the table to discuss their research process. Communities also have standard that measures the same phenomenon using a different form and set of tools. It is important for both parties to identify the good they bring and contribute to research; and make room for fraternizing with the competencies of both groups. We thereby promote the concept of collaboration at the minimum, and partnership as the ultimate goal of the community engagement process.
- We premise the strategies and activities identified for the community engagement process on the notion that science generates facts and so do the interrogations and interactions with community members. Dialogue and engagement of these two – researchers and community members - needs to be on the platform of equity. Both partners need to acknowledge these competency and equality. They therefore need to respect each other’s’ views and perspectives and allow their concepts of ‘right’ jointly influence the design of research protocol. The debates, perspectives, and philosophies of right and wrong should not make parties lose the sense of emergency, urgency, and the needed actionable response during an infectious disease emergency.
- We equally recognize the danger of focusing on an epidemic and its urgency to respond or implement a time-bound research. Such focus distracts from the true ethical issue, which is that little attention is paid to the underlying chronic issues of under-development of research infrastructures and lack of resources for health systems including possible use of research outcomes to improve the health of affected populations. These same issues allow for re-emergence of epidemics, and increases the prospect for poor control of new and re-emergent epidemics in West Africa. Yet, despite this danger, we need to focus response during an epidemic but need to institute measures that allow for sustainable development in worse affected countries as an ethical imperative of the epidemic response.
- It is reasonable to develop context-specific ‘best practice’ examples of stakeholder engagement for a range of different types of research. Thus, community collaboration in clinical trials conducted during an emergency should be, to the extent that is possible, in the context of the particular emergency. Urgency implies the exclusion of the luxury of time. This implies that the breadth and depth of stakeholder engagement as reflected in the GPP-ED should be fast-tracked not that any element of a stakeholder engagement process should be eliminated.
- A stakeholder engagement plan needs to be developed that is driven by evidence generated from the formative research. Research teams should engage multiple people with multiple skills when developing the stakeholder engagement plan, and when implementing the community consultative process. The process should engage all segments of the population – diverse gender, ethnicity, occupation – for the purpose of ensuring inclusion of group-specific concerns in the design and implementation of the research.
- The community engagement plan – a component of the stakeholder engagement plan - should include plans for advocacy, media engagement, external communications, and community mobilization. It should address fear and misconceptions that cause panic when dealing with new disease entities. It may be more challenging to implement a research-focused community engagement plan during an epidemic when the need for instituting much needed public health measures is critical. Where there are conflicts between human, infrastructure, and financial resource needs for research versus public health responses– the public health response should be prioritized above research conduct. All research-related community engagement programmes should be clearly delineated from public health measures.
- One needs to balance the breadth and depth of community engagement efforts during clinical trials in the context of the nature of the emergency. The economy (and political economy) of community engagement in research changes with the nature and phase of a particular emergency. However, no matter the nature and phase of the emergency, community engagement is essential and should not be omitted when conducting clinical trials.
- Community engagement efforts start from research conceptualization through to design, implementation, monitoring, and dissemination. However, community engagement efforts for clinical trials implemented during emergencies often start at the research implementation stage post research protocol approval. This old paradigm of clinical trial conduct needs to change as it is more difficult and challenging to seek protocol and budget modifications if thisis left to this stage. Community should benefit from the research in measureable ways, including through the development of a fair benefits model (employment, capacity building, infrastructural development, etc.). Post-research benefits, including access to developed study products, are equally essential.
- The minimum element of a community engagement plan that should be implemented during an infectious disease emergency irrespective of the ‘acuteness’ of the epidemic, is the engagement of national and local stakeholders, and the public dissemination of information about the research. These engagement efforts should complement the public health response and include efforts to allay fears.
- National and local leaders should be engaged prior to any engagement of community members. Thisprocess should include meeting with national and local leadership, religious and traditional leaders, community health practitioners, and community based organizations,as well as community advocacy groups. This process can create the needed political will and conducive environment for support of the research. This is referred to as the community entry process.
- Engagement with political leaderships as part of the stakeholder engagement plan, should not subsume the goal of engaging community members in research design and conduct. Where the promise of clinical trials becomes appealing to governments in need of a response to address a health crisis, political pressures may result in exclusion of the community engagement element of a stakeholder engagement plan. Political urgency for research conduct and engagement of community members in the design and implementation of the research should be balanced.

We acknowledge that not all forms of ‘political’ leadership may necessarily represent the interests of a wider population. Also, leaders in these roles can exercise power in perverse ways that researchers may not want to support. This is a reason why it is important to have multiple strategies for actively engaging community members so that no one actor/set of actors has/have overall control of this connection between researchers and the wider population.

Community engagement promotes inclusion, equity, and respect. Engagement with trusted community members who act as community gatekeepers is a demonstration of respect. Respect is also shown through acknowledgement of existing structures, acknowledgement of community roles in research, and acknowledgment of the need for collaboration through active listening and open dialogue for successful research. Researchers should help leaders learn about the aim of the research and study procedures. Their views and suggestions for study implementation are important as a local responsiveness measure. We acknowledge that identifying gatekeepers may come with its challenges as there are often multiple layers of gatekeepers of political, security, religious, and cultural importance. The formative research should help identify leaders who are important and central to engendering community trust.

- The community engagement plan should include a comprehensive process that requires researchers to work with identified community leaders to understand the research and make inputs to the research implementation process prior to rigorous implementation of other community engagementactivities. Efforts at seeking community leaders’ support simply so that they can exercise their authority to mobilise community support for the research should be discouraged. Engagement of community leaders should be to enable them to understand the proposed research, ask clarifing questions, make inputs,and faciliate access to other key stakeholders in the communtiy for the purpose of dialoguing prior to research implementation.
- Where there are emergency community needs identified through the community consultative process that may affect disease control, researchers have a moral obligation to help the community resolve the emergency needs. These needs do not have to addressed through direct funding from the research. Researchers can help link the community to appropriate institutions in a position to address its needs. We acknowledge that these needs are often complex and deep seated; and are related to things like gendered power or economic inequities that researchers can’t really resolve. But they can try to limit these: they can make a plan in advance of any new research to address the issues – particularly by setting up and facilitating referral pathways to existing longer term institutional providers where these exist.
- Community engagement programmes should include the wide engagement of the media - local radio, newspapers, TV, public events – to discuss the role of the research during the epidemic so people understand a lot more about the research. Media engagement can lead to fast track public dissemination of factual information, enhanced through holding educational sessions with journalists to clarify possible myths and misconceptions prior to the public engagement process. The media engagement process should be a continuous process. Where human movement and human contact are limited and restricted, extensive engagement of the media with dissemination of factual information is critical.
- Opportunities should be made to provide regular research updates to community members. Due consideration should be given to the suggestions made by community members or their representatives during protocol development. The community should be informed about how their suggestions have been handled. This bi-directional relationship between researchers and community members is a mechanism to institute transparency and accountability on the path of the researchers. It also helps researchers gain broader knowledge on how community understands the research.

Where community ownership of research is the goal, it is best to support, empower, and resource community members to think critically and strategically about the research aims, processes, and implementation plans, promoting community conversations that are grounded in evidence. It is also important that there is a consolidated process that supports leadership and ownership of the community response, and promotes political support of local and national leaders for the purpose of ensuring that the clinical trial complements the ongoing public health response. Research should not in anyway distract from human, infrastructure,and financial resources invested in the public health response.

- Participants who were engaged in phase I trials can serve as educators about the trials and can be part of the team for community mobilisation. This could help community members have a more accurate picture of what participation in the research involves, and help address concerns community members may have.

**3.1. New Questions**

- Information sharing is an important component of community engagement. Should information be segmented by age, gender, ethnicity, nationality of identified stakeholders or other identified parameters?
- The GPP-ED serves as a guide for community engagement during infectious disease epidemics. Is this guide more appropriate or is it more applicable or differentially applicable for use for communitarian societies against an individualist society?
- Do community engagement practices differ between types of research – social science research as oppose to clinical trials? If yes, how should this differ?
- Would a community engagement plan differ if the Principal Investigator of the study was local or if International? We acknowledge that the study context would play a major in deciding this. No stereotyped or one-size-fits--all answer. Community engagement should be tailored to the community’s needs. Would this require deeper inputs if the PI is abroad. What element of the community engagement process will differ?
- When is community engagement the right thing to do for a research? Conversely, can it be the wrong thing? Can it obstruct research, slow down emergency response, distract research, or prevent timely attention to emergency needs?
- Can community engagement in an emergency, like civil liberties, be suspended? What are the harms associated with community engagement in research during an infectious disease epidemic? In what situation then can the harm of community engagement outweigh the good?
- When does research become a political economy? How is risk in trial participation negotiated in the form of payment/compensation? When research with high risk of uncertainty is conducted, does the community engagement process have to address this concern? Is it not an ethical stance to want to take care of your family? Why is it not an ethical consideration to want to brokerage care of the family for a research with a high risk for mortality and severe morbidity? Ethically well-planned research still kills people. So asking for insurance is an ethical demand that should be given legitimate consideration.
- What role can the ethics committee play? Can the competency of laypersons on ethics committees be built so they also play the role of community gatekeepers during the review of protocols to be implemented in infectious disease emergencies? Does the role of laypersons on ethics committees substitute for community engagement during an epidemic?
- Are IRB members community positionalities? Can they allow shifts in community engagement practice during an infectious disease emergency? Can they allow shifts in community engagement practice during any form of research? Can they be made accountable to the communities they serve? Can having IRB develop expertise role in promoting community engagement allow for community omission in research – can IRB substituted for community engagement structures.
- How can ethics governance systems that are efficient during an emergency be developed? Who should be involved in making decisions on the behalf of communities where it is not practical for the wider community/individual patients to be consulted?
- If the practice of ethics is a moving wall? Who determines what participant remuneration should be? Community Advisory Board can make input; while EC approves. However, CABs are often constituted after EC protocol approval as part of the research implementation process. What does this mean in terms of instituting community research support mechanisms for research in general, and research conducted during an emergency?
- Where does the notion of right- correct, fair and just - come in here? Think of the role of variability in perceptions, role of research question, and how community engagement is framed as a right and not just an absolute. What does this imply when we assess the potential for coercion?
- Science is often a slow response to an epidemic and oftentimes, triggered by something – political interest, availability of funds.
- How do you facilitate a genuine community engagement process? How do you ensure ‘objectivity and validity’ of the community engagement process?
- Is there a cascade of aspiration and a cascade of ‘emergenciness’ that needs to be developed? There is great variability in an infectious disease emergency. Outside an infectious disease situation, research design is matched with the research question and competency is the basis for the research design.
- The political economy of community engagement is to sell the research for whatever purpose. How is this reality factored into the community engagement process to ensure every element of coercion is excluded from the engagement process?
- Is time the only an essential element to be considered when considering community engagement practice during an infectious disease epidemic?
- Is there a place for communities to invite other forms of expertise to negotiate their stance during a research protocol development? Would this be appropriate when community representatives seek for extra information rather than legitimate areas for debate being ‘ring fenced’ by researchers.

Acuteness

o f

emergency

Utopian practices

Reality

Community engagement

**4.0. Considerations for research design and implementation**

- Formative research conducted prior to the design of the research implementation plan, using a participatory approach, should highlight historical, social and political concerns that can influence community participation in research. It should also generate information on the concerns of vulnerable community members and how their needs could be addressed while implementing the research. Vulnerability is a contextual concept and should be qualified by an understanding of what the person is vulnerable to and why. In many low resource settings almost everyone in a remote rural setting will be vulnerable to poor health care access.
- Clinical trials should complement and enhance the public health response. Where there is a public health response that supports the compassionate use of research products, thee research design should not exclude the use of such drugs licensed for compassionate use as a public health response measure.

Research conducted during an emergency should at least in part, address the concerns of the affected community. To do this, the affected community needs to be involved with the design and implementation of research in order to influence outcomes that affect them, and to ensure that the outcome will benefit them. Research design and implementation should address local perspectives about the research To achieve these, knowledgeble community members need to be be involved with protocol development. We acknowledge that it can be tricky to identify community members who can give advice on research (some technical experience), reflect the views and values of the wider community (close understanding/connection) and be trusted by the community members (another essential quality bordering on integrity) to serve as community representatives when developing the research protocol. Given these challenges, community accountability - how the wider community can come to know who is taking on this role and how they are fulfilling it - is an issue that needs to be resolved. Accountability measures are also trust building measures.

- Priority should be given to research focused on understanding how disease transmission could be prevented in the future, and those research that explore how to mitigate the impact of the diseases in the present.
- There is no urgency for clinical trials conduct during an emergency phase of an infectious disease epidemic with high mortality and morbidity unless there is a clinical response to be tested or implemented. Research should not distract from public health response and research that compliments the public health response should be prioritized.
- Implementation research should be prioritized during the acute phase of an epidemic with high fatality. Implementation research seeks to explore improved or novel ways to address disease conditions or systems and to enhance quality and quantity of disease management. Approach which increases the prospect for inclusion of all affected populations and does not exclude anyone from accessing therapy (with known and unknown efficacy) should be prioritized for implementation.
- Investigative research should be limited at the acute phase of an infectious disease epidemic with high mortality and morbidity like the case of the Ebola epidemic as it will distract from resources – human especially – that should otherwise be invested in the public health response. Investments in the epidemic should be prioritized to save all lives and not to prioritise the future by sacrificing some in the present.
- Clinical trials studying the efficacy of drugs that may have outcome of immense benefit for the patients should be prioritized during an emergency. In effect, phase II and III drug trials could be prioritized in the acute phase of the infection. Trials trying to establish the safety profile of a therapeutic agenda (phase I studies) are not of immense priority in an infectious disease epidemic with high mortality and morbidity rates like that of the West Africa Ebola epidemic.
- Research that does not diminish the prospect for morbidity and mortality arising from the infectious disease emergency cannot be prioritized.
- Research conducted during a self-limiting infectious disease epidemic should be flexible enough to address immediate community needs that decrease mortality and morbidity. The use of an adaptive trial design to test interventions increases prospect for possible changes to be made to trial designs in ways that the trial also responds to community needs.
- Where the epidemic affects children and pregnant women with as much severity as other members of the community, the trial should be planned in a cascaded fashion that allows for the recruitment of children and pregnant women during different waves of the trials. Considerations for the study of research interventions in children and pregnant women need to be contextualized from the research concept development.
- Research is never an emergency even in an emergency situation. We recognize the importance of conducting research for a disease that little is known about. For a disease with a burn-out period, and sporadic rare episodes like Ebola, the sense of urgency implies that processes needs to be fast-tracked; no research process should be omitted neither should the quality of any of the processes compromised.
- Individuals who volunteer as research participants do so as selfless citizens who believe in the benevolence of the state. Their participation is based on a conviction of erradicating the disease or developing a drug for future that would maintain the public health safety to all. There is also an assumption that the state would take care of their needs in the face of any eventualities. In states where such benevolence is not available, then insurance cover becomes the stop gap. Altruism is however, not the main motivating factor in most studies conducted during outbreak especially one associated with intense fear and uncertainty. Research tends to be used as a resource-seeking strategy (<https://www.ncbi.nlm.nih.gov/pubmed/25302444>). The state would take care of the needs of research participants in case of any mortality or morbidity. In states where such benevolence is not available, then insurance cover becomes the stop gap.

**4.1. New questions**

- The need to design studies using reproducible methods that will generate results that can be validated by peers limits researchers exploring new and uncharted research methodologies. How can alternative clinical trial designs that respect community nuances be validated for trial product registration?

**5.0. History, political and socio-cultural context of research conducted during emergency**

- When research is situated in a context of political distrust, low research literacy, and poverty, exploitation, coercion and undue inducement is possible.
- Formative research conducted prior to study design and implementation needs to create historical and socio-cultural maps: an honest appraisal of how the community is constituted. In the absence of literature on the community, engage privileged academics and other elites who are members, or connected to members of the affected community who can provide the needed information.
- While multi-centre studies make significant efforts at developing study protocols that makes the study outcomes generalisable to all irrespective of geographical locations, the nuances of the study protocol implementation needs to address and respect the local nuances – their culture, norms, value, sreligion and practices. This does not compromise the scientific validity of the research and it enhances its ethical integrity. These are great consideration and possible challenges that need to be overcome when protocols are developed and driven by research team not familiar with the nuances of the communities where they plan to implement the research.
- The communication plan developed should influence beliefs grounded in people’s experience. This should not be considered unimportant. Engagement and re-engagement with the community is important for change to happen.
- The notion of rationality is important. History helps you understand the rationale for what communities consider as important. History can provide grounds for understanding the context of actions, perspectives and expectations from research. Experiences with disease control, experiences with respect or abuse of human rights, proximity to disease and proximity to death all influence the understanding of the context for research in emergency situations, and attitudes towards community engagement. History of past unethical trials and the socio-economic context of people influence this rationality, and will shape individual and shared perspectives on research design and community mobilization process. The context of the lives of FSW Cambodia influenced the context of their engagement with the PrEP trial, so also does the contest of the lives of community members influence the way they engage with research.
- Stigma of populations also occurs during research and this can have damaging impact. It is important to prevent this possibility.
- Research itself is about writing a history. Historical context of a research influences the design and implementation of current and future research. The history and legacy researchers leave behind in the community they work also has the potential to make future research conduct in that community more challenging or more feasible. When community engagement processes are handled poorly, collateral damage is done and it affects the entire research enterprise. This puts a responsibility on the researcher to exercise care in the conduct of research. Researchers need to be responsible to the community of researchers.

The research design must consider community's traditions and believes about tropical and traditional medical practices. Traditional values may vary from one community to another. It is erroneous to presume that (geographic) communities are homogenous. While there may be different constituents with particular interests that should be taken care of, groups that are vulnerable in different ways need to be identified and supported to alleviate the potential impact of the epidemic resulting from their vulnerability.

**5.1. New questions**

- There is a relationship between empathy and ‘emergenciness’ – the severity of emergencies can sometimes have a distancing of ‘dehumanising’ aspect. We need to ask how do the things we value in human traits shift with the ‘emergenciness’ of an epidemic? How do we locate beneficence and benevolence in a cascade of epidemics and the local context of the disease? And how does this play out in a communitarian or a neoliberal society or other forms of social structures?
- How do you vulnerability in the context of an infectious disease epidemic to which everyone is at equal risk?

**6.0. Community omission**

- The forms of community engagement will differ per the context of the epidemic including logistical barriers associated with community engagement processes. Lower intensity of community engagement may be permissible in researchers with lower risk In clinical trials however, there may be a higher requirement to include community concerns and consideration in the component risk/benefit analysis before taking a decision on the merit of the research.
- Community omission is acceptable when the pattern and epidemiology of the disease is poorly understood and the community affected is undefined. Once a community is identified, engagement with the community should commence. Face to face community engagement programmes can be omitted when safety of individuals is highly compromised through contacts. safety concerns should not preclude community engagement programmes through use of appropriate strategies.
- Community omission is not encouraged irrespective of the nature of the epidemic emergency. While it can be argued that in an emergency, civil and other liberties can and are suspended for the greater good, we argue that community exclusion (an active conscious action of exclusion) for the benefit the greater good will breed resistance and contempt for science processes; and it’s a prelude for research failure.
- The time allocated for the community engagement process may be shortened during an emergency. The magnitude of the epidemic should not affect the depth of each component of the community engagement practice.
- There are multiple reasons why community exclusion can happen. These include lack of skills by researcher, history of failure when engaging the community, and researchers who do not believe in it. These are not justifiable reasons for community exclusion.

**6.1. New questions**

- Read about ways and practices about knowing and the rationalization for knowing. The rationalization thinking and characterize knowing; and the more individualistic and less universal way of knowing. Where does community engagement and community mobilization lie in this context of learning?
- All stakeholders have different ways of ‘knowing’. Does the community need to know about the science of the research in the same way that the scientists or researchers do?
- In research conducted during emergencies, what is the balance between the priorities of science, its methods, it outcomes and community development?
- Is community omission also feasible for social science and not just clinical trials? Does community omission in social science research about omission in research planning rather than omission from field work?
- What is and how is the allowable clinical trial related risk conducted during an infectious disease emergency determined?
